# Extraction and characterization of spherical nanocellulose from sesame husks

**DOI:** 10.1016/j.heliyon.2024.e41269

**Published:** 2024-12-16

**Authors:** Mohammed Al-haql, Hoda Habbal, Bassam Al Oklah, Nesreen Qurabi

**Affiliations:** a Department of Food Science, Faculty of Agriculture, Damascus University, Syria; b Department of Food Technology, National Commission for Biotechnology, Damascus, Syria; c Department of Food Engineering Technologies, Faculty of Technical Engineering, Aleppo University, Syria

**Keywords:** Sesame, Husks, Acid hydrolysis, Nanocellulose, Eco-friendly

## Abstract

The objective of this study was to extract and characterize nanocellulose from sesame husks, which are typically discarded as waste by sesame processing facilities. However, these husks are rich in cellulose, presenting a valuable potential source for nanocellulose. Sesame husk cellulose (SHC) was initially isolated through a multi-step process that removed oil, hemicellulose, and lignin. Sesame husk nanocellulose (SHNC) was subsequently obtained via acid hydrolysis. Energy-dispersive X-ray (EDX) analysis revealed a purity of 99.32 % for SHNC. The yields of SHC and SHNC were 25.16 % and 9.17 %, respectively. SHNC exhibited a lower surface charge (−27.2 mV) compared to SHC (−15.5 mV). FTIR confirmed the presence of characteristic cellulose bands. Dynamic light scattering (DLS) revealed average particle diameters of 2235 nm for SHC and 108.1 nm for SHNC. Atomic force microscopy (AFM) and field-emission scanning electron microscopy (FE-SEM) analyses showed that SHNC particles were spherical to oval-shaped, with average diameters of 78.41 nm and 74.30 nm, respectively. The crystallinity index was higher for SHNC (67.74 %) compared to SHC (41.02 %). Thermogravimetric analysis (TGA) indicated greater thermal stability for SHC (TMax 317 °C) compared to SHNC (TMax 287 °C). These results demonstrate the potential of sesame husks as a sustainable and valuable source of nanocellulose.

## Highlights

• Sesame waste to nanocellulose (SHNC): A Sustainable Approach.
• Cost-effective extraction method developed, eliminating the need for dialysis.
• SHNC exhibited a spherical to oval-shaped, suspension stability and high purity.
• SHNC showed good thermal stability, though slightly lower than that of SHC.
• SHNC is a versatile nanomaterial suitable for a wide range of applications.

## Graphical abstract

**Figure:**
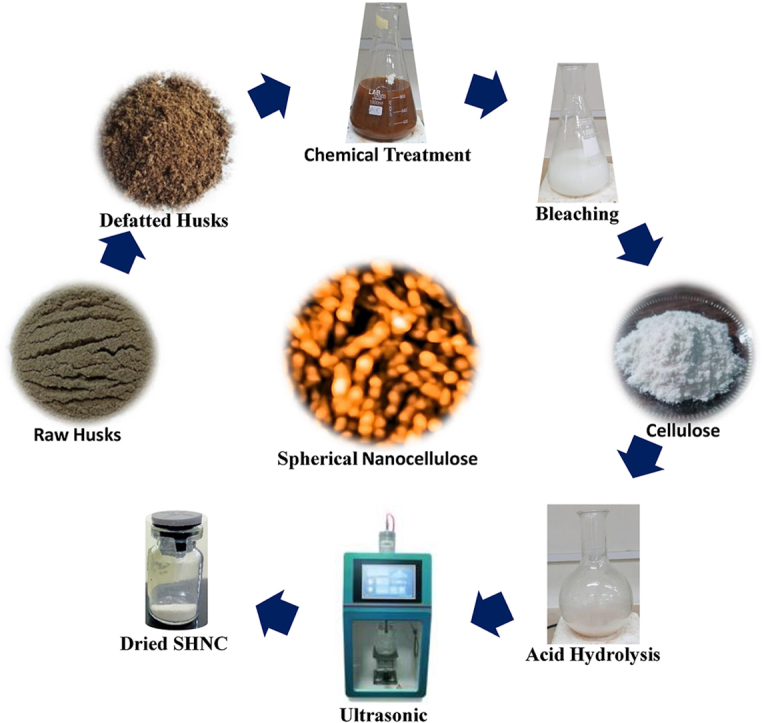

## 1 Introduction

Sesame (*Sesamum indicum*) plays an important role in human nutrition; its seeds are used to produce oil, halva, bakery products, and a variety of other foods [1]. Sesame processing discards 15–18 % of the seed weight as husks, which are often neglected as waste [2,3]. This is considerably higher than the 2 % hulls generated by soybean processing [4]. Sesame husks are a valuable source of various nutrients, containing 10.23 % protein, 42.03 % dietary fiber, 12.21 % oil, and 23.90 % ash [1]. Notably, their high cellulose content 31–54 % [3], which is higher than the 29 % found in pea hulls [5], makes these husks promising candidates as a renewable source of cellulose.

Cellulose is a high-molecular-weight polysaccharide consisting of linear chains of D-glucopyranose units (cellobiose) linked by β-(1 → 4) glycosidic bonds [6]. Traditionally, wood and cotton have been the primary sources of cellulosic fibers. However, increasing environmental concerns and the depletion of forest resources due to rising demand for wood have led to a growing interest in non-wood cellulosic materials [7]. Recently, agriculture waste, such as rice straw and corncob [8], wheat straw and barley [7], soy hulls [4], pea hulls [5], banana peels [9], have been studied as the biomass resources for producing nanocellulose.

Plant nanocellulose refers to cellulosic materials isolated from plant cellulose fibers with at least one dimension smaller than 100 nm [10]. It can be classified into two main categories: cellulose nanoparticles and cellulose nanofibers. The first is prepared via acid hydrolysis of pure cellulose, while the second is obtained through mechanical disintegration using high shear forces [11].

Nanocellulose exhibits remarkable properties, including biodegradability, non-toxicity, renewability, optical transparency, low density (1.6 g/cm³), a large surface area (250–500 m^2^/g), high tensile strength (7.5–10 GPa, 10 times that of steel), an elastic modulus of approximately (100–150 GPa), and a low axial thermal expansion coefficient of 10⁻⁷ K⁻^1^. Additionally, the presence of numerous hydroxyl groups makes it highly reactive and amenable to chemical modification, further expanding its potential applications [[12], [13], [14]].

As a result, nanocellulose finds applications in diverse fields, such as reinforcing synthetic polymers (elastomers, thermosets, or thermoplastics) to produce lighter and more corrosion-resistant materials [15]. It is also used to improve the properties of edible films and smart food packaging [5,7]. In the medical field, nanocellulose-based hydrogels can serve as drug carriers, regulating drug release rates in the body [10], and is used for the preparation of antimicrobial wound dressings and tissue engineering scaffolds [7]. Additionally, it can function as a Pickering stabilizer in the food, cosmetic, and pharmaceutical industries [16].

The rod-like and whisker shapes are the most prevalent forms of cellulose nanoparticles, with widths ranging from 4 to 70 nm, lengths from 100 to 600 nm, and a crystallinity index of 54–88 %. Recently, a new class of cellulose nanoparticles with spherical to oval-shaped and diameters of 80–120 nm has emerged [6,8]. The specific shape, size, and other properties of each cellulose nanoparticle class depend on several factors, including the cellulose source [6], hydrolysis time, temperature, acid type, and concentration [8].

The shape of cellulose nanoparticles significantly influences their properties and potential applications. Spherical nanoparticles are particularly well-suited for a wide range of applications, particularly in the medical, food, and water treatment [8]. Doan and Chiang [17] further emphasize the superior properties of spherical nanoparticles compared to rod-like nanoparticles, due to their uniform shape, narrow size distribution, large surface area.

Zhang et al. [18] were the first to successfully produce spherical nanocellulose from commercial cellulose with a size of 470 ± 100 nm using a combined approach of mixed acid hydrolysis and ultrasound. Spherical nanocellulose was also prepared from cotton fibers using a combination of enzymatic hydrolysis and ultrasound, with a size range between 100 and 526 nm [16].

A previous study successfully extracted nanocellulose from sesame husks with dimensions ranging from 235 to 342 nm, exhibiting a spherical to oval shape. This was achieved using acid hydrolysis (35 % H_2_SO_4_, 1:30 g/mL, 50 °C, 180 min) followed by high-pressure homogenization [19]. However, the particle size was relatively large, and the acid hydrolysis time was prolonged. Additionally, most of the properties of these particles were not investigated.

In this study, spherical to oval-shaped nanocellulose particles were successfully extracted from sesame husks with high purity and nanoscale dimensions using a simple and cost-effective method. Various techniques were employed to characterize these particles, investigating their chemical composition, surface charge, morphology, size, functional groups, crystallinity index, and thermal stability in order to evaluate their suitability for future applications. This approach could potentially reduce environmental pollution, lower the costs associated with disposing of these husks, and promote the upcycling of agricultural and industrial waste into high-value materials, thus contributing to a more sustainable future.

## 2 Materials and methods

### 2.1 Materials

White sesame husks were obtained from Ayyam Zaman Company (Damascus countryside, Syria) and were prepared according to the method outlined by Elleuch et al. [1]. Our previous study reported the chemical composition of these husks: 94.92 % dry matter, 8.09 % oil, 9.63 % protein, 30.17 % ash, and 52.10 % carbohydrates (including 35.97 % dietary fiber) [20]. N-hexane, sulfuric acid (H2_S_O_4_, 98 %), and sodium chlorite (NaClO_2_, 80 %) were purchased from Sigma-Aldrich Co. (St. Louis, MO, USA). Hydrochloric acid (HCl, 37 %) and sodium hydroxide (NaOH, 99 %) were obtained from Schatlau Co. (Barcelona, Spain).

### 2.2 Extraction of cellulose from sesame husks

Cellulose extraction from sesame husks was adapted from the methods of Purkait et al. and Zhang et al. [3,19], with minor modifications. To remove oils and waxes, 100 g of sesame husks were initially soaked in a 1:4 (w/v) hexane solution for 16 h at room temperature. The mixture was then filtered, the solvent was recovered, and the remaining residue was dried at 105 °C. The defatted husks were subsequently subjected to acid treatment with 1 N hydrochloric acid (1:10 w/v) for 1 h at 80 ± 5 °C with continuous stirring. The residue was washed with dH_2_O and dried at 105 °C. In the second step, an alkaline treatment was performed using a 5 % sodium hydroxide solution (1:15 w/v) at 80 ± 5 °C with continuous stirring for 3 h. The residue was then washed with distilled water and dried at 105 °C. Finally, the remaining residue underwent bleaching with a 1 % sodium chlorite (1:15 w/v) solution at a pre-adjusted pH of 4.5 using 2 % acetic acid. The mixture was then heated to 80 ± 5 °C for 3 h with continuous magnetic stirring. The resulting residue was washed with distilled water, dried at 105 °C, ground in a laboratory mill, and sieved through 100 μm to obtain pure cellulose. The product was designated as SHC (sesame husks cellulose).

### 2.3 Preparation of nanocellulose

Nanocellulose was prepared using the acid hydrolysis method according to Purkait et al. and Zhang et al. [3,19], With minor modifications. One gram of SHC was mixed with 15 mL of 57 % (wt) H_2_SO_4_ at 45 °C with continuous stirring for 60 min. To quench the reaction, cold dH_2_O was added. The resulting mixture was centrifuged for 15 min at 9000 rpm, and the precipitate was washed with dH_2_O and resuspended several times until the pH reached 1–1.5. The dialysis separation process for sulfate ions and other soluble materials was replaced by dispersing the colloidal suspension in 1 L of dH_2_O and leaving it at refrigerator temperature for 48 h. It was then resuspended and washed with dH_2_O and the precipitate was collected, at which point the pH reached 5.5–6. The precipitate was re-dispersed in 100 mL of dH_2_O and the pH was adjusted to 7 using NaOH (0.5 N). Finally, the colloidal suspension was subjected to ultrasound in an ice water bath for 30 min [5]. The colloidal suspension was dried using an Alpha 1–2 LD plus freeze dryer from CHRIST, Germany. The product was designated as SHNC (sesame husks nanocellulose).

### 2.4 Determination of yield

The yield of SHC and SHNC was determined following the method of Romruen et al. [8].

### 2.5 Characterization of SHC and SHNC

#### 2.5.1 Determination of ash

The ash content was determined using AOAC method No. 942.05 [21].

#### 2.5.2 Determination of diameter and zeta potential

The average diameter, particle size distribution, and zeta potential of SHC and SHNC were measured using dynamic light scattering (DLS) with a Malvern Zetasizer Nano ZS instrument (UK) at 25 °C. The sample was diluted 100 times with dH_2_O and sonicated for 5 min before analysis [13].

#### 2.5.3 Field emission scanning electron microscopy (FE-SEM)

The dried SHNC was examined using (FE-SEM) a MERA3 LMU instrument (TESCAN, Brno, Czech) at an accelerating voltage of 20 kV. Prior to FE-SEM analysis, a thin layer of gold was sputtered onto the sample using an ion sputter coater with low deposition. Energy-dispersive X-ray (EDX) integrated with the FE-SEM was employed for elemental analysis of the SHNC [22].

#### 2.5.4 Atomic force microscopy (AFM)

AFM (EasyScan2 Flex AFM, Nanosurf, Leistal, Switzerland) was employed to characterize the topography and morphology of the SHNC in tapping mode. Surface roughness was quantified using Nanosurf Report Expert v 5.0 software. Prior to AFM analysis, a 0.01 % (w/v) aqueous suspension of the sample was deposited on a glass slide and allowed to air-dry at room temperature [22].

#### 2.5.5 Image analysis

The dimensions (diameter) of over 200 SHNC particles were determined using image analysis with ImageJ 1.46 software (NIH, USA) from FE-SEM and AFM images [23].

#### 2.5.6 Fourier transform infrared (FTIR)

The functional groups of SHC and SHNC were identified using a FTIR spectrometer (FTIR-4200, Jasco, Japan). The dried samples were ground and pressed into discs with (KBr) pellets (1:100). The FTIR spectra were recorded in the range from 4000–600 cm^−1^ [3].

#### 2.5.7 Thermal analysis

The thermal stability of SHC and SHNC was evaluated using a thermogravimetric analyzer (TGA) (TG 50, Mettler Toledo, Switzerland). The experimental parameters were as follows: temperature range, 30–700 °C; heating rate, 10 °C/min; nitrogen gas flow, 100 mL/min; sample weight, 8 mg [3].

#### 2.5.8 X-ray diffraction (XRD)

The X-ray diffractograms of SHC and SHNC were produced by an X-ray diffractometer (Philips X-ray, Amsterdam, Netherlands) under the following conditions: voltage, 40 kV; current, 30 mA; X-ray source, CuKα with a wavelength of 1.54056 Å, 2θ scan range, 5–89°, detector, Linear PSD monochromator, germanium. The crystallinity index (CrI) was determined using the Segal equation as described by Romruen et al. [8].

## 3 Results and discussion

### 3.1 Extraction and yield

Cellulose was isolated from sesame husks through a multistep pre-treatment process. This involved removing oils and waxes, followed by acid treatment with hydrochloric acid to eliminate acid-soluble components and weaken the chemical bonds in the lignocellulosic matrix [24]. The first and second stages resulted in weight losses of 7.10 ± 0.21 % and 21.32 ± 1.67 %, respectively. Alkaline treatment led to a further 32.42 ± 2.19 % weight loss due to the dissolution of hemicelluloses and alkali-soluble materials. A 6.2 ± 0.81 % weight loss was observed during the bleaching stage, attributed to the removal of color and lignin [19]. The observed differences in weight loss between this study and previous investigations on sesame husk cellulose [3,19], are attributed to variations in the extraction method. The residual ash content in the SHC after processing was 7.82 ± 0.11 %, significantly lower than that of the raw husks, which had 30.17 % [20]. This reduction is attributed to the removal of mineral elements or the formation of soluble salts during pre-treatment, particularly hydrochloric acid treatment. The alkaline nature of sesame husk ash, with calcium accounting for approximately 10 % of the dry mass [1], supports this explanation.

**Fig. 1:**
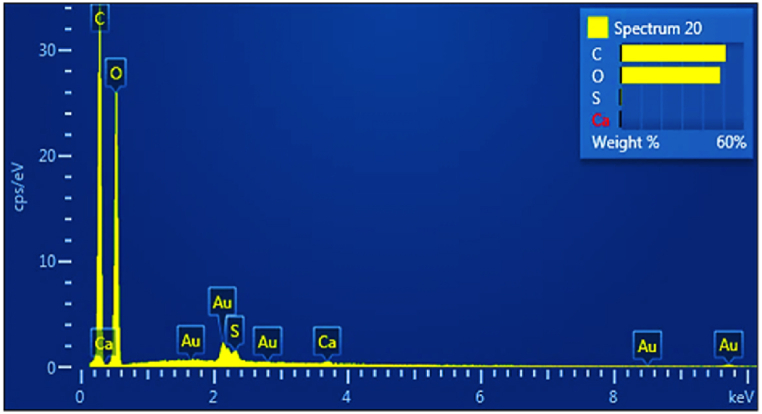
EDX spectra of SHNC.

SHNC exhibited a remarkably low residual ash content of 0.86 ± 0.11 % after acid hydrolysis. This high purity was further confirmed by energy-dispersive X-ray (EDX) analysis, which revealed that carbon and oxygen were the dominant elements, accounting for 99.32 % of the SHNC composition, as shown in Table 1 and Fig. 1. Trace amounts of calcium and sulfur were also detected, the latter attributed to the formation of sulfate esters during acid hydrolysis [25].

**Table 1:** EDX analysis of SHNC.

Element	Wt (%)	Atomic (%)
Carbon (C)	51.08	58.35
Oxygen (O)	48.24	41.37
Sulfur (S)	0.52	0.22
Calcium (Ca)	0.16	0.05
Total	100	100

This study significantly reduced residual sulfur content compared to previous dialysis-based methods. Raza et al. [13] and Kumar et al. [22] reported 5.7 % and 0.72 % sulfur in nanocellulose from palm fibers and sugarcane bagasse, respectively. Our value of 0.52 % is lower but still slightly exceeds the 0.37 % reported by Kassab et al. [12] for sunflower cake nanocellulose. The sequential dispersion and washing steps used for SHNC in this study effectively removed sulfuric acid residues, providing a cost-effective alternative to dialysis.

Yield, a crucial factor in determining the economic viability of acid hydrolysis, is directly influenced by process parameters such as time, temperature, and acid concentration [28]. The yields of SHC and SHNC were 25.16 ± 0.71 % and 9.17 ± 0.89 %, respectively. These results were consistent with the findings of Zhang et al. [3] for sesame husk cellulose, and the low yield of SHNC was attributed to the loss of mineral salts and amorphous cellulose during acid hydrolysis. Notably, the obtained yields were higher than those reported for tomato peels 2 % [14], banana peels 5 % [9], soybean hulls 8 % [4], and jackfruit peels 7 % [26]. However, it was lower than the yield of nanocellulose extracted from 20 % cotton waste [16], as yield depends on the source of cellulose [7].

### 3.2 Average particle diameter and zeta potential

**Fig. 2:**
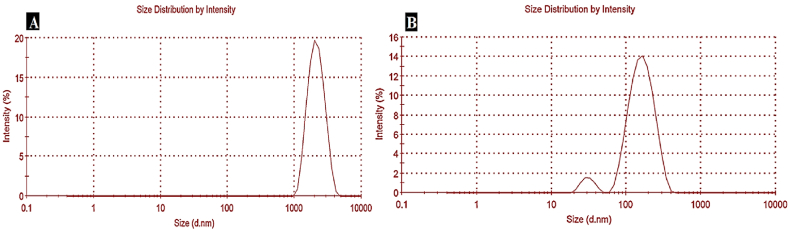
Particle size distribution of SHC (A) and SHNC (B).

A single peak with an average diameter of 2235 nm was observed in the DLS analysis of SHC, as shown in Fig. 2 (A) and Table 2. The polydispersity index (PDI) was 0.305, indicating a monodisperse size distribution [13]. Notably, the average diameter in this study was lower than the 3480–4210 nm range reported by Durmaz et al. [27], for sesame husk cellulose. This difference was due to the pre-treatment with hydrochloric acid, which resulted in smaller particle sizes and a reduced ash content compared to laboratory methods that employed only alkaline treatment and bleaching under optimal extraction conditions. The latter method led to cellulose particle agglomeration and grinding difficulties, resulting in larger particle sizes.

**Table 2:** Particle size, polydispersity index (PDI), and zeta potential of SHC and SHNC.

sample	Size (nm)	PDI	Zeta potential (mV)
SHC	2235	0.305	−15.5
SHNC	108.1	0.229	−27.2

On the other hand, the SHNC depicted in Fig. 2 (B) exhibited a bimodal distribution with two peaks at diameters of 25.02 and 138.6 nm, respectively. The average diameter was 108.1 nm as shown in Table 2. The presence of these two peaks indicates that there are two different particle sizes within the sample. This can be attributed to the initial milling process of SHN, which resulted in particles of varying dimensions despite sieving with a 100-μm sieve [25], as well as the random degradation of cellulose during the acid hydrolysis process [28]. The PDI was 0.229, indicating a narrow particle size distribution. For polymer-based nanomaterials, a PDI value of ≥0.2 is generally considered favorable [13]. The obtained results were lower than those reported by Purkait et al. [19], due to the differences in acid concentration and hydrolysis time.

Zeta potential is a measure of suspension stability, indicating the degree of repulsion between similarly charged adjacent particles in the suspension [9].

**Image 1:**
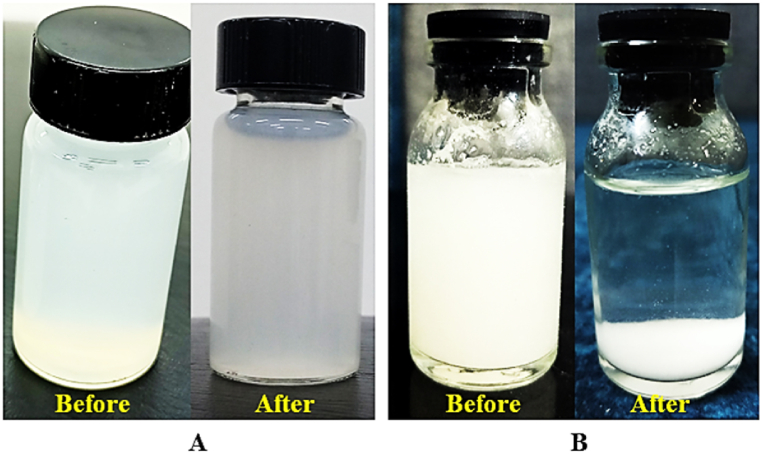
Suspension stability of SHCN (A) and SHN (B).

Table 2. Presents the zeta potential values at pH 7 for SHC and SHNC, which were −15.5 and −27.2 mV, respectively. Additionally, Image 1 (A), taken at time zero and after 8 weeks of storage at refrigerator temperature, demonstrates the stability of the SHNC suspension, which remained bluish-white in color and exhibited no sedimentation even after 3 months. In contrast, complete sedimentation of the milky white SHC suspension occurred after only 4 h, as shown in Image 1 (B). This difference in stability was due to the variations in particle size, shape, and surface charge [17]. Banerjee et al. [29] reported that the particles with surface charges between 0 and -15 mV are considered low or uncharged, leading to suspension instability. The low negative charge of SHNC was attributed to negatively charged sulfate ions formed during sulfuric acid hydrolysis, which generate repulsive forces that hinder aggregation and promote suspension stability [30]. The zeta potential and suspension stability of SHNC in this study were consistent with those reported for spherical nanocellulose from cotton and palm fibers, with zeta potential values of −27 and −27.7 mV, respectively [13,31].

### 3.3 Morphologic analysis and dimensionality

**Fig. 3:**
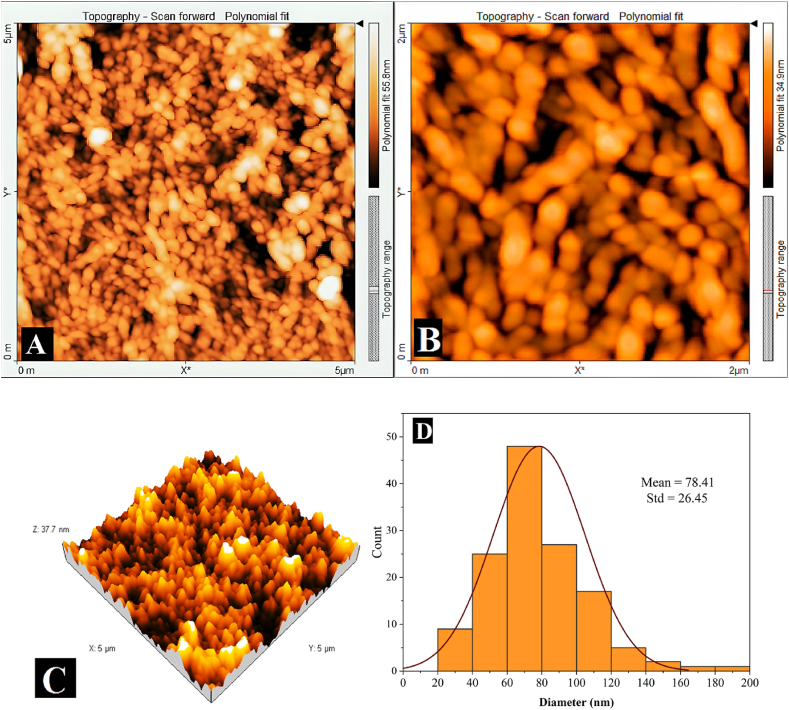
AFM images of SHNC morphology in 2D: (A) 5 μm scale, (B) 2 μm scale, (C) 3D topography, (D) Particle size distribution.

Atomic force microscopy (AFM) was used to study the surface topography, shape and distribution of SHNC particles. Images were taken in both D2 and D3 dimensions, as shown in Fig. 3 (A, B, C). The results revealed a surface roughness (arithmetical mean height,Sa) of 16.7 nm and a root mean-square height (Sq) of 19.9 nm over a 5 × 5 μm^2^ area. The particles appeared in a spherical shape and others were oval. The observed agglomerates in the AFM images of SHNC can be attributed to the drying process of the SHNC suspension on the surface of the examination slide. An annular pattern emerges because evaporation rates differ across the droplet. The evaporation rate at the edge of the droplet is faster than at the center, causing the liquid and particles to flow towards the periphery. Consequently, particle deposition is more pronounced at the edge, leading to the formation of agglomerates [32]. Fig. 3 (D) presents the particle size distribution obtained from AFM images. The average diameter of the particles was 78.41 ± 26.45 nm.

**Fig. 4:**
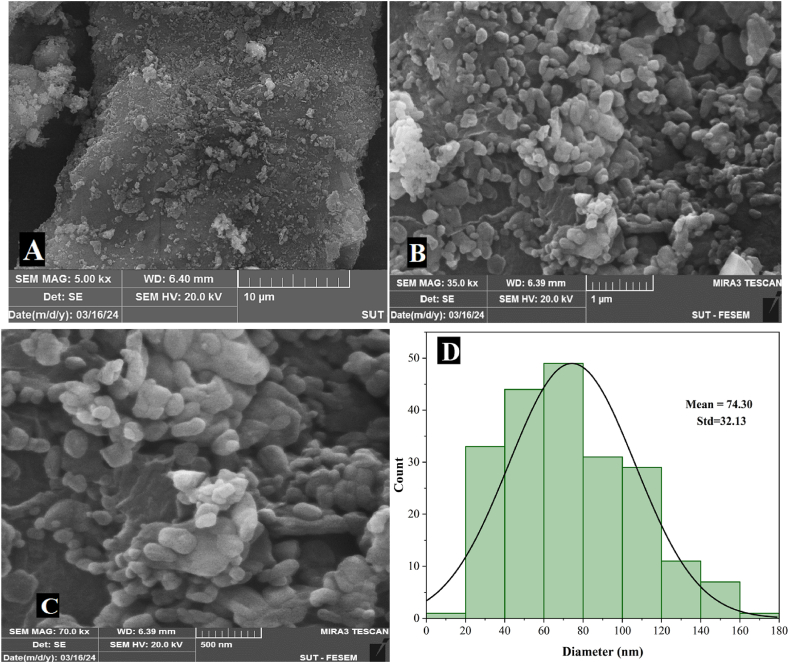
FE-SEM images of SHNC at different magnifications: (A) 10 μm, (B) 1 μm, (C) 500 nm, (D) Particle size distribution.

In addition to the aforementioned characterization, the morphology and distribution of SHNC particles were investigated using FE-SEM at different magnifications. Fig. 4 (A, B, C) reveals that, at a magnification of 10 μm, some particles appeared as individuals, while others were agglomerated. At a higher magnification of 1 μm, and 500 nm, the particles exhibited spherical to oval-shaped, while others had irregular shapes, with some particles exhibiting agglomeration. The observed variations in particle shape and size were attributed to the random degradation of cellulose during the acidic hydrolysis process [28,32].

As for the agglomerates, this was due to the formation of hydrogen bonds between individual particles; the hydrophilic nature of cellulose promotes the self-assembly and aggregation of these particles due to the intermolecular attraction mediated by hydrogen bonds [33]. Furthermore, the freeze-drying process employed for sample preparation prior to microscopy can also contribute to agglomerate formation [34]. As evidenced by the particle size distribution histogram derived from FE-SEM images in Fig. 4 (D), they were on the nanoscale, with diameters between 17.2 and 165.16 nm. The majority of particles fall within the 60–80 nm range, with an average diameter of 74.30 ± 32.13 nm.

This observation was consistent with AFM results, but differed in particle dimensions obtained using the Zeta size. This discrepancy arises from the fact that the Zeta sizer works on the principle of dynamic light scattering (DLS). In this case, the DLS tends to mask the scattering intensity of smaller particles by the stronger scattering intensity of larger particles. Consequently, not all smaller particles are detected [35]. Particle size is also influenced by sample preparation conditions for analysis and measurement techniques [32]. Our findings were consistent with those of a previous investigation on sesame husk nanocellulose in terms of morphology but differ in particle dimensions [19]. It was considerably smaller in this study. The results also correspond to those of Li et al. (2022) [31], for the extraction of spherical nanocellulose from cotton, where particle diameters ranged from 20 to 180 nm with an average of 78 nm.

### 3.4 Functional group identification

The FTIR analysis shows similar fingerprints for both the SHC and SHNC. However, there were slight differences in intensity at specific wavelengths, likely due to the pre-treatment and acid hydrolysis processes [3].

**Fig. 5:**
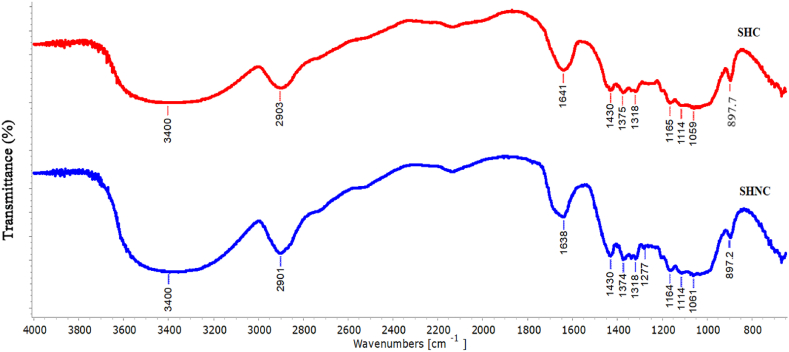
FTIR spectra of SHC (in red) and SHNC (in blue).

As shown in Fig. 5, a broad peak was observed in both spectra at 3400 cm⁻^1^, corresponding to the stretching vibration of -OH groups that form hydrogen bonds between and within cellulose molecules [36]. A peak also appeared at 2901 cm^−1^ in SHC and 2903 cm^−1^ in SHNC, which is related to the symmetrical vibration of the C-H [3]. Additionally, peaks at 1638 cm^−1^ and 1641 cm^−1^ for SHC and SHNC, respectively, were attributed to the OH bending vibration associated with the presence of water (moisture) in the samples [37].

The peak at 1430 cm^−1^ corresponds to the bending vibration of the -CH_2_ [3]. While the peaks observed at 1375 cm^−1^ and 1318 cm^−1^ for both SHC and SHNC are attributed to the C-H and the C-O-C bond, respectively [38,39], the peaks at 1164,1114, and 1064 cm^−1^ for SHC and 1159, 1114, and 1061 cm^−1^ for SHNC were assigned to the C-C and C-O-C in the anhydroglucose ring [17,38].

The characteristic peak for the β-(1 → 4) glycosidic linkage in cellulose appeared at 897 cm^−1^, in agreement with previous findings [3,39]. Notably, the peaks observed in the FTIR spectra of both samples indicate the presence of cellulose I, suggesting that the cellulose structure remained stable during acid hydrolysis [17,36].

A comparison of the FTIR spectra reveals the appearance of a small peak at 1277 cm^−1^ in the SHNC spectra, attributed to the C-O-S bond vibration of sulfate ester groups formed during acid hydrolysis [5]. Furthermore, the absence of peaks at 1745 cm⁻^1^ and 1502 cm⁻^1^ indicates the successful removal of non-cellulosic components (such as lignin and hemicellulose) during pre-treatment [3].

### 3.5 XRD diffraction analysis

**Fig. 6:**
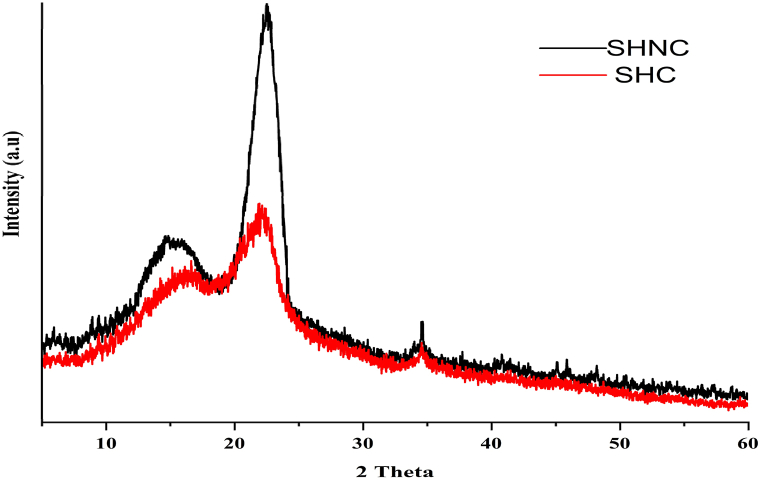
XRD pattern of SHC (in red) and SHNC (in black**)**.

The XRD patterns of both samples (Fig. 6) showed the characteristic peaks of cellulose I [3]. These peaks appeared at 15.5°, 22.5°, and 34.5°, corresponding to the (101), (002), and (004) crystallographic planes of cellulose [40]. Furthermore, the SHNC pattern displayed a strong peak, particularly at 22.5° (used for calculating the CrI). This suggests a higher degree of crystallinity in SHNC compared to SHC [3]. The crystallinity of cellulose, quantified by the CrI (%), was 41.02 % for SHC. Notably, acid hydrolysis significantly increased the crystallinity of SHNC, with the CrI increasing by 34.85 % to reach 67.74 %. This improvement is due to acid preferentially dissolving the amorphous regions, thereby enriching the relative content of crystalline regions [7,13].

Our findings were consistent with the observations reported by Durmaz et al. [27], for raw and acid-treated sesame husks cellulose, they obtained CrI of 42.2 % and 68.9 %, respectively. However, the CrI values in this study were higher than the 60 % reported by Zhang et al. [3]. Conversely, the CrI of the SHNC was higher than that of nanocellulose extracted from rice husks 66.3 % [7], sunflower cake 62 % [12], and pistachio hulls 58.6 % [41]. However, it was lower than the CrI of nanocellulose extracted from microcrystalline cellulose, 74.11 % [40]. This is because the CrI of nanocellulose is influenced by the source and the chemical and mechanical treatment methods [7]. The XRD results were supportive and consistent with the FTIR results in terms of cellulose type and crystallinity.

### 3.6 Thermal stability analysis

**Fig. 7:**
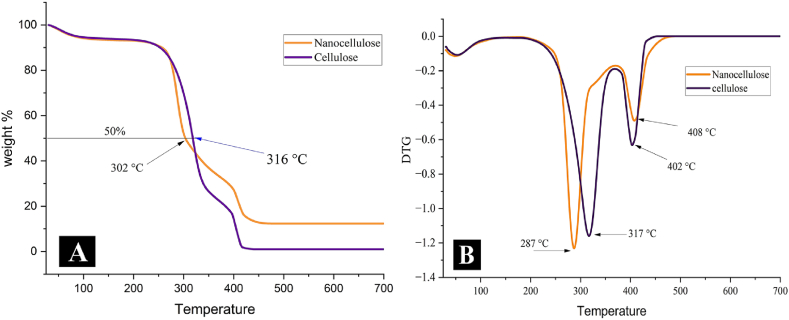
TGA (A) and DTG (B) curves of Cellulose and Nanocellulose from Sesame Husks.

The results of the thermogravimetric analysis (TGA) and differential thermogravimetry (DTG) are presented in Fig. 7, while Table 3 summarizes the thermal degradation behavior for both samples. As shown in Fig. 7(A and B), weight loss occurred in three stages for both samples. In the first stage, there was a decrease of 5.56 % and 5.69 % for SHC and SHNC, respectively, between 30 °C and 150 °C. This initial weight loss is due to the evaporation of adsorbed moisture on the surfaces of the samples as well as chemisorbed and H-bonded water molecules in the samples [33,37].

**Table 3:** Thermal degradation results of SHC and SHNC from sesame husks.

stage	H_2_O	Main stage				carbonic residue degradation		
samples	weight loss (%)	Onset (°C)	T Max1 (°C)	End (°C)	weight loss (%)	T Max2 (°C)	weight loss (%)	residue at 700 (°C)
SHC	5.56	251	317	380	73.31	402	19.05	1.45
SHNC	5.69	243	287	374	62.35	408	19.68	12.91

The second stage represents the main phase of thermal degradation. It is characterized by a gradual weight loss, starting at 251 °C and 243 °C, respectively, for SHC and SHNC, and continuing until 380 °C and 374 °C. The maximum degradation temperature (Tmax) was 317 °C and 287 °C for SHC and SHNC, respectively. This stage was accompanied by a weight loss of 73.31 % and 62.35 % for SHC and SHNC, respectively, as shown in Table 3. The weight loss in this stage is attributed to two primary concurrent processes: cellulose dehydration (water evaporation) and depolymerization. Depolymerization involves the cleavage of glycosidic bonds, forming free radicals (glycosyl units) and volatile compounds such as furfural and acetaldehyde. Primarily, levoglucosan is formed, which subsequently decomposes into gases like carbon dioxide (CO₂), carbon monoxide (CO), hydrogen (H₂), and light hydrocarbons such as methane (CH₄), ethylene (C₂H₄) and ethane (C₂H₆), and the formation of a charred residue [22,[42], [43], [44]].

As observed, SHNC shows lower thermal stability compared to SHC. This is attributed to the increased rate of heat transfer due to its nanoscale size, and large surface area [33,41]. Additionally, the sulfate ester groups present on the surfaces of nanocellulose play a catalytic role in facilitating the degradation and depolymerization of SHNC, leading to a decrease in activation energy [12,30]. Furthermore, the thermal stability of nanoparticles is significantly influenced by their size and shape [45].

In this study, the thermal degradation of SHC and SHNC occurred within the temperature range reported by previous studies for sesame husk cellulose, and nanocellulose of wheat and rice straw [3,7,27].

Fig. 7 (A, B) and Table 3 illustrate the third stage of weight loss, involving the decomposition of carbon residues. The TMax values were 402 and 408 °C for SHC and SHNC, respectively, with corresponding weight losses of 19.05 and 19.68 %. The rapid degradation of carbon residues at higher temperatures leads to the abrupt weight loss, releasing more CO and flammable gases such as CH4, C₂H₄, and C₂H₆ [42,44].

The char residue content was significantly higher in SHNC (12.91 %) than in SHC (1.45 %) at 700 °C. This can be attributed to the abundance of free hydroxyl groups in SHNC, the presence of flame-retardant sulfate ester groups [25], and the high carbon content of SHNC due to its high crystallinity, which leads to increased char formation [33]. These findings are consistent with the results reported by Zhang et al. [3].

## 4 Conclusion

Cellulose (SHC) was successfully extracted from sesame husks using a multi-step process. Sulfuric acid hydrolysis was used to obtain nanocellulose particles (SHNC), which exhibited desirable properties, including good suspension stability, nanoscale dimensions, crystallinity, and good thermal stability. The sequential dispersion and washing steps employed for SHNC in this study proved to be effective in removing sulfuric acid residues, offering a cost-effective alternative to the expensive dialysis method. The transformation of sesame husks into nanocellulose provides a multifaceted solution that addresses waste management challenges and promotes the development of sustainable nanomaterials, eco-friendly with diverse applications. Future research should explore the potential applications of SHNC in various fields, such as polymer-based nanomaterials, drug delivery, and food additives. Additionally, optimizing the extraction process to enhance SHNC yield and particle size control is warranted.

## CRediT authorship contribution statement

**Mohammed Al-haql:** Writing – original draft, Methodology, Formal analysis, Data curation. **Hoda Habbal:** Validation, Supervision, Project administration. **Bassam Al Oklah:** Visualization, Validation, Supervision. **Nesreen Qurabi:** Writing – review & editing, Resources, Methodology, Investigation.

## Ethics statement

This research adheres to all relevant ethical standards, guidelines, and regulations. It does not involve any experiments on humans or animals.

## Data share statement

Data will be made available on request.

## Funding

This study was funded by 10.13039/501100020595Damascus University, Syria. funder No. 501100020595.

## Declaration of competing interest

The authors declare that they have no known competing financial interests or personal relationships that could have appeared to influence the work reported in this paper.
